# Preoperative assessment of cognitive function and risk assessment of cognitive impairment in elderly patients with orthopedics: a cross-sectional study

**DOI:** 10.1186/s12871-020-01096-6

**Published:** 2020-08-01

**Authors:** Shuyuan Gan, Yang Yu, Jiateng Wu, Xiaodong Tang, Yueying Zheng, Mingcang Wang, Shengmei Zhu

**Affiliations:** 1 Department of Anesthesiology, the First Affiliated Hospital, College of Medicine, Zhejiang University, No. 79 Qingchun Road, Hangzhou, Zhejiang China; 2 Department of Anesthesiology, Taizhou Hospital, Wenzhou Medical University, Linhai, 317000 Zhejiang China

**Keywords:** Preoperative, Elderly, Orthopedics, Cognitive impairment, Risk factors

## Abstract

**Background:**

Preexisting cognitive impairment is emerging as a predictor of poor postoperative outcomes in seniors. Nevertheless, cognitive impairment in a large proportion of geriatric patients has not been well identified and diagnosed.

**Methods:**

This is a cross-sectional study. Mini-mental state examination scale was used to assess the cognitive function of elderly patients aged ≥65 years undergoing orthopedic surgery preoperatively. The baseline, living habits and laboratory examination results of two groups were compared, and a multivariable logistic regression model was used to identify independent predictors of preoperative cognitive impairment.

**Results:**

A total of 374 elderly patients with orthopedic surgery indications met the inclusion criteria, and 28.61% of them had preoperative cognitive impairment. Multivariable logistic regression analysis showed that age (OR = 1.089, *P < 0.001*), subjective sleep disorders (OR = 1.996, *P = 0.021*), atherosclerosis (OR = 2.367, *P = 0.017*), and high cholesterol level (OR = 1.373, *P = 0.028*) were independent risk factors for preoperative cognitive impairment, while high education level performed as a protective factor (compared with the illiterate group, primary school group: OR = 0.413, *P = 0.009*; middle school or above group: OR = *0.120*, *P < 0.001*).

**Conclusions:**

The prevalence of preoperative cognitive dysfunction in geriatric elective orthopedic surgical patients was high. Our study identified venerable age, low level of education, subjective sleep disorders, atherosclerosis, and high cholesterol level as risk factors for preoperative cognitive impairment in these patients. Understanding these risk factors contributes to assisting in prevention and directed interventions for the high-risk population.

## Background

More than 300 million people worldwide undergo major surgery each year, and approximately 1 in 3 surgical procedures are performed on those ≥65 years old [1]. The elderly are often complicated with more than one underlying disease preoperatively, and surgical outcomes tend to be poor in such patients with a higher rate of postoperative complications, including persistent organ dysfunction [2, 3]. Preoperative evaluation of the vital organ systems has been a routine surgical preparation for decades, especially for the elderly [4]. Early detection of organ impairment helps provide information for perioperative care planning [5].

Good brain health plays an important role in medical needs and functional recovery. There are several reasons to believe that the evaluation of brain function is crucial in elders about to undergo surgery. First, brain dysfunction is common in the elderly. Survey studies have shown that 5–10% of elderly patients aged ≥65 years in the community have dementia. Once mild cognitive impairment (MCI) is fully considered, the prevalence of cognitive disorders is up to 35–50% [6, 7]. Then, a significant proportion of cognitive impairment, particularly at the stage of MCI, goes undetected clinically [8]. Last but not the least, delirium is arguably one of the most important postoperative complications, affecting 20–80% of patients older than 65. Among these surgical patients, preexisting cognitive impairment, as well as an increased duration of surgery and receiving a general anesthetic, are associated with an increased risk for postoperative delirium (POD) and other surgical outcomes [9–11]. Conversely, clinical interventions, such as interoperative infusion of dexmedetomidine, are protective factors [12, 13]. Diagnosis of preoperative cognitive impairment enables early identification of at-risk patients and therefore timely management of postoperative cognitive complications to reduce the occurrence [14].

Due to insufficient clinical staffing, unclear evaluation methods, lack of objective records, and insufficient understanding of the disease, the evaluation of preoperative cognitive function of elderly patients has not been classified as a routine project at home and abroad [15]. Mini-mental state examination (MMSE) is the most commonly used cognitive function test scale, can be used as a screening for epidemiological investigations, and is recommended for the evaluation of the preoperative cognitive status of elderly patients [16]. Moreover, the Chinese Medical Association Geriatrics Branch recommended MMSE to evaluate the preoperative cognitive status of elderly patients in 2016 [17].

The aim of this study was, therefore, to explore the prevalence of preoperative cognitive impairment in patients ≥65 years old with MMSE and to examine the association of cognitive impairment with preoperative risk factors in an older population scheduled for orthopedic surgery.

## Methods

### Ethical approval

All procedures performed in this study involving human participants were in accordance with the ethical standards of the institution (The Clinical Research Ethics Committee from the First Affiliated Hospital, College of Medicine, Zhejiang University. The reference number: 900 on 10th August, 2018). All patients provided written informed consent for the publication of any associated data.

### Patients

This was a cross-sectional study and was completed in a manner consistent with the STROBE statement. The participants included in the current analysis were all patients scheduled for orthopedic surgery at our institution and were recruited between August 2018 to June 2019. Patients were included if they were 65 years of age or order with ASA I-III, and underwent elective orthopedic surgery. Exclusion criteria were patients who underwent surgical treatment within 6 months and conditions that prevented participation in the assessment, such as limitations in visual, hearing and dominant hand ability, no surgical plan, and refusal to follow up.

### Data collection

Risk factors that have been epidemiologically defined in this perioperative setting were measured [7, 18]. All participants were asked to complete a standardized set of self-report questionnaires. Demographic characteristics were recorded, including age, sex, height and weight for body mass index (BMI), degree of education, smoking and drinking status, widowed or divorced, exercise (≥4 times per week) and subjective sleep quality (well or not). Comorbidities were recorded to calculate the Charlson Comorbidity Index (CCI), as well as the presence of known neuropsychiatric diagnoses. Primary diagnosis and prehospital current psychotropic medication use (yes/no) were also recorded.

Homocysteine (Hcy), albumin (ALB), alanine aminotransferase (ALT), triglyceride (TG), total cholesterol (TC), low density lipoprotein (LDL) and fasting blood glucose (FBG) were measured and collected on the basis of clinical need, but at least on the preoperative day.

### Neuro-psychologic testing

MMSE is an effective screening tool for various degrees of cognitive impairment, including MCI and dementia, assessing the domains of attention and concentration, executive functions, memory, language, visuoconstructional skills, conceptual thinking, calculation and orientation. The total score is 30 points; the higher the score, the better the cognitive function. Considering the impact of education level on MMSE assessment, combined with the actual situation in China and previous studies, the thresholds for those who were illiterate or attended at most primary school, or middle school were ≤ 17, ≤20, and ≤ 24, respectively [19, 20]. Individuals with a score below the threshold value were considered as cognitively impaired.

In this way, patients were divided into cognitive normal and cognitive impairment groups. In our study, the score for each domain and the overall score were recorded. A single researcher who was trained in the use of the tool prior to recruitment performed all of the cognitive screening patient interviews.

### Statistical methods

We calculated descriptive statistics. Categorical variables were summarized as frequencies and proportions, normally distributed continuous variables were expressed as the mean (standard deviation, SD), and nonnormally distributed continuous variables were expressed as median (interquartile range, IQR). An unpaired t-test was used to test for normally distributed continuous variables, the Mann–Whitney U-test was used for variables without normal distribution, and the chi-square test was applied for categorical data as appropriate.

A logistic multivariable regression model was performed to screen independent risk factors for predicting preoperative cognitive impairment. Variables with a significant difference of *P < 0.1* in the univariate analysis were deliberately included in the following logistic multivariable analysis model to identify independent risk factors.

Differences were considered to be statistically significant if the *P < 0.05* (two-tailed). Statistical analysis was performed using SPSS version 23.0 (IBM Corporation, Armonk, NY, USA).

## Results

**Fig. 1:**
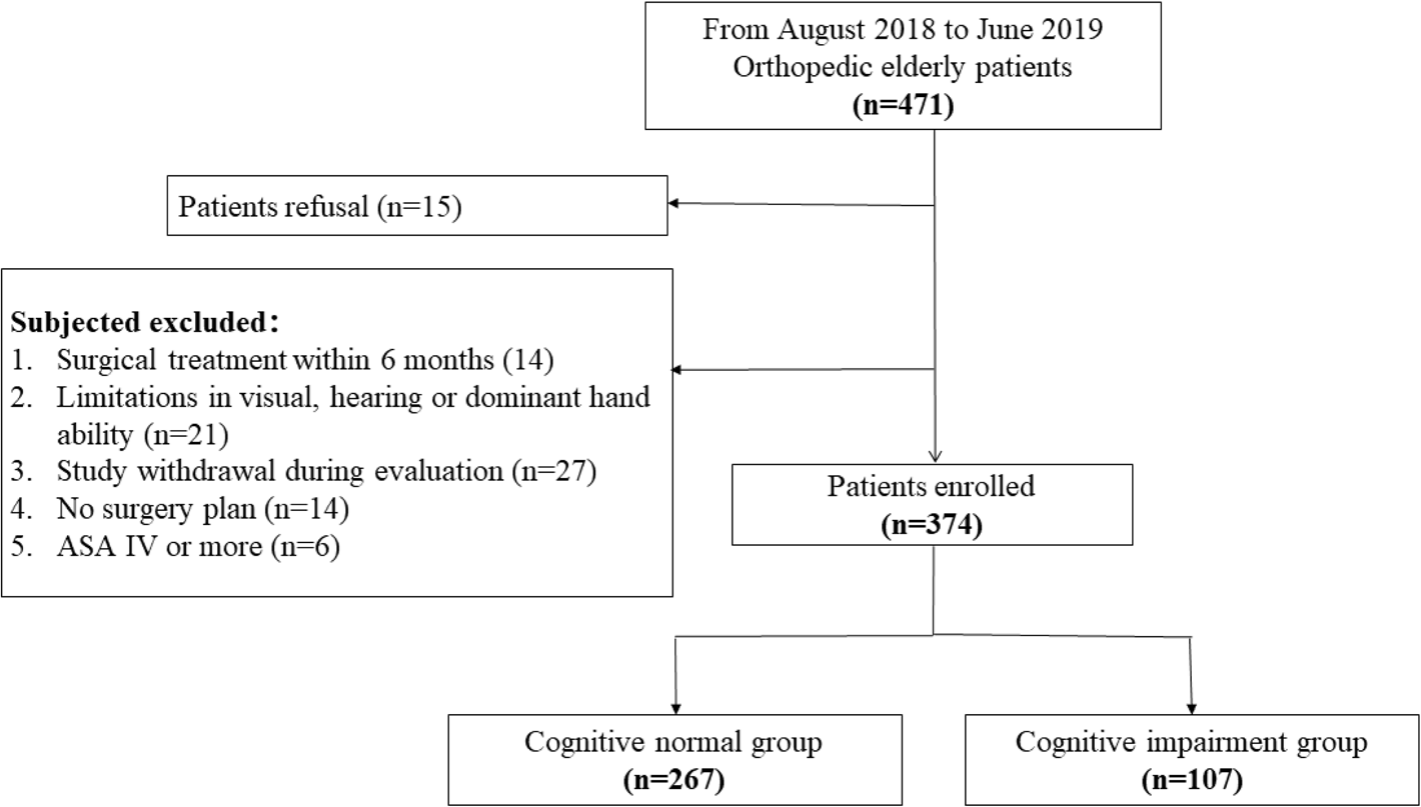
Cohort recruitment diagram of patient enrollment, follow-up, exclusion and analysis

Data were available from 471 patients with questionnaire and cognitive testing. Figure 1 shows a patient flow diagram. The number of patients completing follow-up neuro-psychologic testing preoperatively was 374. The reasons for exclusion were patient refusal (15), study withdrawal during evaluation (27), no surgery plan (14), surgical treatment within 6 months (14), limitations in visual, hearing or dominant hand ability (21), and ASA IV or more (6). Preoperative cognitive impairment was diagnosed in 107 (28.61%) patients according to the assessment of MMSE.

### Baseline parameters and preoperative characteristics

**Fig. 2:**
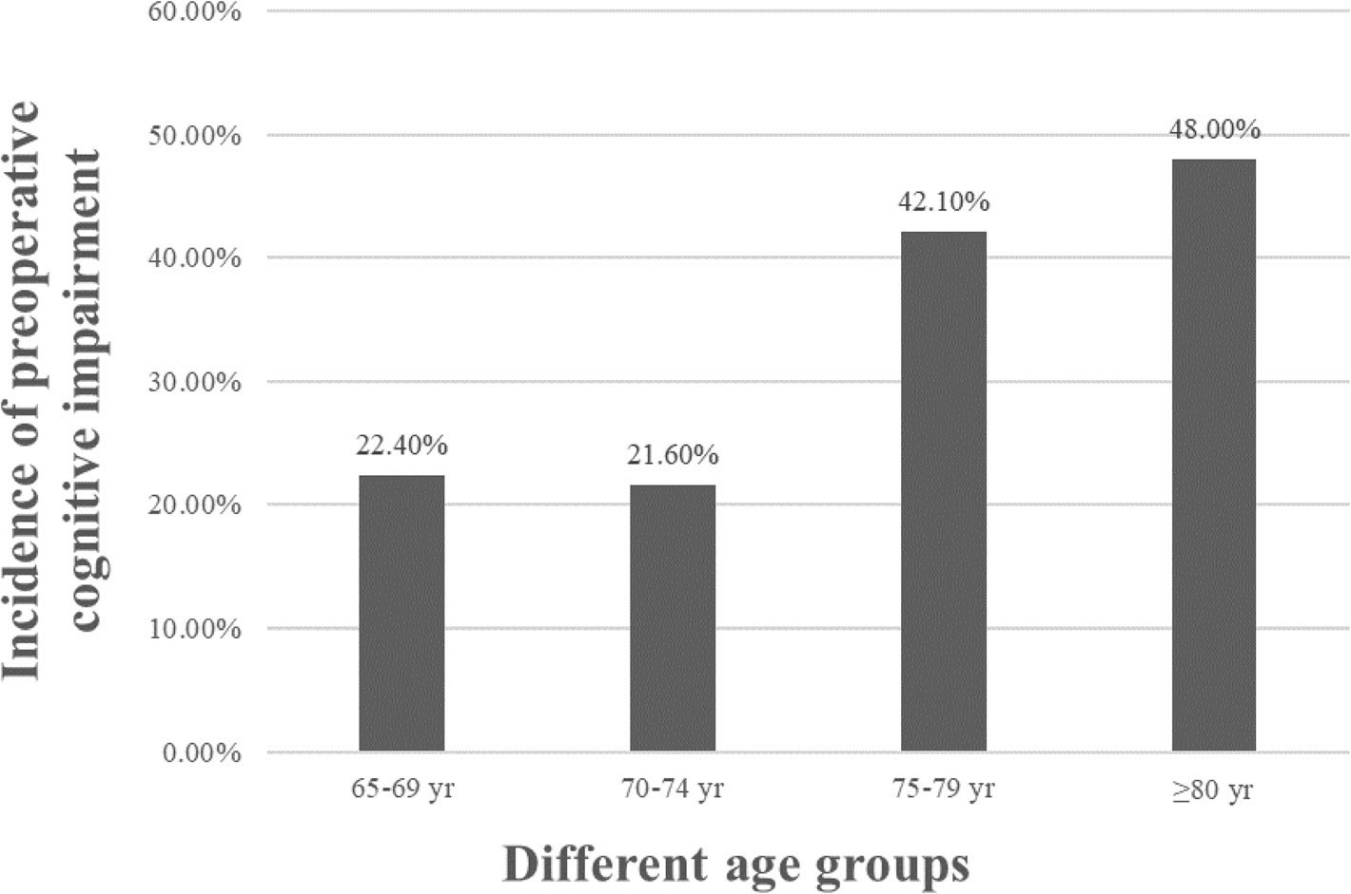
The prevalence of cognitive impairment in different age groups

The characteristics of participants are shown in Table 1. The median age with IQR was 70 (68, 75.25) yr in all participants; 72 (68, 76) yr and 70 (67, 74) yr in females and males, respectively, showing a statistical significance (*P < 0.001*). Notably, the prevalence of cognitive impairment in different age groups is shown in Fig. 2, and increased significantly with age. A total of 53.2% of patients were admitted to the hospital for spinal lesions, 29.7% for hip, knee or tibia lesions, and 17.1% for other parts, in which there was no difference between patients with and without cognitive impairment.

**Table 1:** Demographic and clinical characteristics of patients with or without cognitive impairment

Variables	Cognitive normal (***n*** = 267)	Cognitive impairment (***n*** = 107)	Statistical values	***P***
**MMSE, median (IQR)**	25 (23–27)	16 (13–19)		
**Age (yr), median (IQR)**	70 (67–74)	73 (68–79)	3.930^a^	< 0.001
**Female, n(%)**	128 (47.9)	73 (62.8)	12.643^b^	< 0.001
**BMI (kg m**^**−2**^**, mean ± SD)**	23.46 ± 3.48	23.00 ± 3.52	1.154^c^	0.249
**Widowed or Divorced, n (%)**	24 (9.0)	15 (14.0)	2.069^b^	0.150
**Level of education**			30.505^b^	< 0.001
**Illiteracy, n (%)**	46 (17.2)	43 (40.2)		
**Primary school, n (%)**	125 (46.8)	50 (46.7)		
**Middle school or above, n (%)**	96 (36.0)	14 (13.1)		
**Comorbidities**				
**Hypertension, n (%)**	162 (60.7)	62 (57.9)	0.237^b^	0.626
**Diabetes mellitus, n (%)**	49 (18.4)	28 (26.2)	2.854^b^	0.091
**Hyperlipidemia, n (%)**	74(27.7)	22 (20.6)	2.049^b^	0.152
**CHD, n (%)**	19 (7.1)	8 (7.5)	0.015^b^	0.903
**CNS disease, n (%)**	32 (12.0)	12 (11.2)	0.044^b^	0.835
**Atherosclerosis, n (%)**	49 (18.4)	30 (28.0)	4.301^b^	0.038
**ASA III, n (%)**	46 (17.2)	33 (30.8)	8.496^b^	0.004
**CCI, median (IQR)**	4 (3–5)	4 (4–5)	1.978^a^	0.048
**Lesions**			0.889^b^	0.641
**Spinal, n (%)**	142 (53.2)	57 (53.3)		
**Hip, knee or tibia, n (%)**	82 (30.7)	29 (27.1)		
**Other parts, n (%)**	43 (16.1)	21 (19.6)		

*MMSE* Mini-mental: state examination, *BMI* Body mass index, *ASA* American Society of Anesthesiologists, *CCI* Charlson comorbidity index, *SD* Standard deviation, *IQR* Interquartile range

^a^*Z* values; ^b^*χ*^*2*^ values; ^c^*t* values

Compared to those without cognitive impairment, subjects with probable or possible preoperative cognitive impairment were older [70 (67–74) vs 73(68–79); *P < 0.001*], more likely to be female (47.9% vs 62.8%; *P < 0.001*), and to have a lower education level (illiteracy group, 17.2% vs 40.2%; primary school group, 46.8% vs 46.7%, middle school group or above group 36% vs 13.1%; *P < 0.001*). Our data showed that a higher proportion of persons with cognitive impairment had atherosclerosis (18.4% vs 28.0%; *P = 0.038*). Cognitive impairment prevalence was not different among patients with hypertension (*P = 0.626*), coronary heart disease (*P = 0.903*), diabetes (*P = 0.091*), hyperlipidemia (*P = 0.152*), and central nervous system diseases (*P = 0.626*) compared with those patients with normal cognition. Patients with cognitive impairment had a higher CCI score than patients without cognitive impairment [4(4–5) vs 4(3–5), *P = 0.048*], and subjects with an ASA score of 3 were more likely in the impairment group than in the normal group (30.8% vs 17.2%; *P = 0.004*). Table 1 describes the variables in more detail.

Patients in the impairment group had a higher level in Hcy (*P = 0.046*), TC (*P = 0.016*) and FBG (*P = 0.041*). Nevertheless, no significant difference was found between the two groups with reference to ALB, ALT, TG and LDL. Table 2 describes the variables of the preoperative serological index in more detail.

**Table 2:** Laboratory test results of patients with or without cognitive impairment

Variables	Cognitive normal (***n*** = 267)	Cognitive impairment (***n*** = 107)	Statistical values	***P***
**Hcy (**μmol**/L), median (IQR)**	11.3 (9.8–13.8)	12.3 (9.9–15.7)	1.991^a^	0.046
**TG (mmol/L), median (IQR)**	1.31 (0.97–1.78)	1.23 (0.96–1.66)	−0.738^a^	0.460
**TC (mmol/L, mean ± SD)**	4.22 ± 0.94	4.48 ± 1.05	−2.431^b^	0.016
**LDL (mmol/L), median (IQR)**	2.45 (1.98–2.94)	2.37 (1.88–2.97)	− 0.480^a^	0.631
**FBG (mmol/L), median (IQR)**	4.89 (4.45–5.45)	5.06 (4.63–5.99)	2.043^a^	0.041
**ALB(g/L), median (IQR)**	42.0 (38.7–44.7)	41.3 (38.5–43.4)	−0.720^a^	0.472
**ALT (U/L), median (IQR)**	15 (12–21)	15 (10–22)	−0.591^a^	0.555

*Hcy* Homocysteine, *TG* Triglyceride, *TC* Total cholesterol, *LDL* low density lipoprotein, *FBG* Fasting blood glucose, *ALB* Albumin, *ALT* Alanine aminotransferase, *SD* Standard deviation, *IQR* Interquartile range

^a^*χ*^*2*^ values; ^b^*Z* values

### Living habits and cognitive functions

In univariate analysis, there was a strong association between subjective sleep disorders and cognitive impairment. Of the 107 participants who developed cognitive impairment, 39.3% had sleep dysfunction at home, whereas of the 267 with normal cognition, 22.8% had sleep dysfunction at home (*P = 0.001*). No significant difference was observed in prehospital psychotropic medication, exercise, cigarette smoking, and history of alcohol consumption of > 5 years between the two groups (Table 3).

**Table 3:** Living habits of patients with or without cognitive impairment

Variables	Cognitive normal (***n*** = 267)	Cognitive impairment (***n*** = 107)	χ^**2**^ values	***P***
**Subjective sleep disorders, n (%)**	61 (22.8)	42 (39.3)	10.303	0.001
**Smoking > 5 years, n (%)**	54 (20.2)	19 (17.8)	0.296	0.586
**Drinking > 5 years, n (%)**	73 (27.3)	24 (22.4)	0.959	0.327
**Pre-hospital psychotropic medication, n (%)**	21 (7.9)	7 (6.5)	0.193	0.660
**Exercise ≥ 4 times per week, n (%)**	106 (40.6)	32 (31.4)	2.658	0.103

### Risk factors for preoperative cognitive impairment

The variables that showed an association with preoperative cognitive impairment (*P<0.1*) were enrolled in the logistic multivariable analysis, including sex, age, education level, subjective sleep disorders, diabetes mellitus, atherosclerosis, ASA score of 3, CCI score, and levels of Hcy, TC and FBG. The results are shown in Table 4. Multivariable regression analysis demonstrated that age (OR = 1.089, 95%CI: 1.037–1.144, *P < 0.001*), subjective sleep disorders (OR = 1.996, 95%CI: 1.112–3.581, *P = 0.021*), atherosclerosis (OR = 2.367, 95%CI: 1.169–4.794, *P = 0.017*), and high level of TC (OR = 1.373, 95%CI: 1.035–1.820, *P = 0.028*) were independent risk factors of cognitive impairment. Conversely, in comparison to illiterate group, higher education levels appeared to be protective of cognitive impairment (primary school group, OR = 0.413, 95%CI 0.213–0.799, *P = 0.009*; middle school group or above group OR = 0.120, 95%CI: 0.052–0.280, *P < 0.001*).

**Table 4:** Logistics multivariable analysis of factors associated with preoperative cognitive impairment

Variables	β	OR	95% CI	***P***
**Age**	0.085	1.089	1.037–1.144	< 0.001
**Sex**	0.192	1.212	0.659–2.229	0.536
**Education level (compared to illiterate group)**				
**Primary school**	−0.885	0.413	0.213–0.799	0.009
**Middle school group or above**	−2.118	0.120	0.052–0.280	< 0.001
**Subjective sleep disorders**	0.691	1.996	1.112–3.581	0.021
**Diabetes mellitus**	−0.325	0.723	0.295–1.770	0.477
**CCI**	0.010	1.010	0.848–1.202	0.913
**ASA**	0.089	1.093	0.520–2.298	0.815
**Atherosclerosis**	0.862	2.367	1.169–4.794	0.017
**FBG**	0.160	1.173	0.957–1.438	0.125
**TC**	0.317	1.373	1.035–1.820	0.028
**Hcy**	0.046	1.047	0.984–1.113	0.145

*ASA* American Society of Anesthesiologists, *CCI* Charlson comorbidity index, *FBG* Fasting blood glucose, *TC* Total cholesterol, *Hcy* Homocysteine, *OR* Odds ratio, *CI* Confidence interval

Taking into account the positive impact of education on MMSE, we conducted a subgroup analysis to further discuss the differences in age, sleep quality, atherosclerosis and TC of people with different education levels. Our data showed that no significant difference was observed in these aspects (*P > 0.05*, Table 5).

**Table 5:** Subgroup analysis of age, sleep quality, atherosclerosis and TC of participants with different education levels

Variables	Illiterate	Primary school	Middle school or above	Statistical values	***P***
**Age (yr), median (IQR)**	70 (67, 76)	70 (68, 75)	71 (68, 76)	1.561^a^	0.458
**Subjective sleep disorders, n (%)**	28 (35.1)	46 (26.3)	29 (26.4)	0.900^a^	0.638
**Atherosclerosis, n (%)**	20 (22.5)	32 (18.3)	27 (24.5)	1.716^a^	0.424
**TC (mmol/L, mean ± SD)**	4.28 ± 1.01	4.4 ± 0.88	4.14 ± 1.08	2.382^b^	0.094

*SD* Standard deviation, *IQR* Interquartile range, *TC* Total cholesterol

^a^*χ*^*2*^ values; ^b^*F* values

## Discussion

The results of this study demonstrate that many geriatric elective surgical patients do poorly on cognitive screening tests preoperatively. Specifically, 28.61% of patients ≥65 years old scored in a range that suggests probable cognitive impairment.

### Preexisting cognitive impairment preoperatively

The prevalence of cognitive impairment among older patients is high, while frequently undiagnosed before admission. Of the 374 patients included, 107 (28.61%) were identified as having cognitive impairment in this study, lower than previous literature reported. Studies have shown that the prevalence of cognitive impairment is as high as 35–50% in community-dwelling older persons, including mild cognitive impairment (MCI) as well as dementia [6, 18]. The prevalence in elderly patients in surgical wards varies with the disease. A study including 152 subjects 60 yr of age and older who were scheduled for total hip joint replacement surgery and undergone preoperative assessment found that 22% were classified as having MCI [21]. The remarkably high prevalence of preoperative MCI in 70% of vascular surgery patients is a cause for concern, among which 88% were undiagnosed before admission [8]. These studies confirm that preoperative mild cognitive deficits are common in older individuals undergoing major surgery.

Nevertheless, routine preoperative evaluation of cognition continues to be overlooked in clinical practice today. Numerous clinical studies have confirmed that preoperative cognitive impairment in older patients undergoing elective surgery has significant impact on postoperative recovery. Lee et al. investigated 129 patients undergoing lumbar spine surgery and found a high prevalence of undiagnosed cognitive impairment (38%), which was associated with a higher rate of POD and prolonged hospital stays [22]. In another retrospective study of 82 older patients undergoing elective spinal surgery, Owoicho et al. found that patients with cognitive impairment were more likely to require an additional stay at a skilled nursing or acute rehabilitation facility [23]. In an observational retrospective study of 1258 patients aged older than 69 years undergoing hip surgery, the severity of cognitive impairment was a prognostic factor for mortality and functional recovery [24]. Greater mortality risk was consistently associated with cognitive impairment before cardiac surgery in a study of 5407 patients with 11 year follow-up [9].

In addition, from June to November 2018, similar papers were published in six well-known journals, suggesting that perioperative neurocognitive disorders (PND) were used to describe the decline or change of cognitive function during the perioperative period to replace postoperative cognitive dysfunction (POCD), which not only extends the timeline of perioperative cognitive follow-up but also emphasizes the importance of preoperative cognitive assessment [25–30].

### Clinical risk factors for preoperative cognitive impairment

The size and function of the brain decrease with age, causing cognitive decline [31]. Our multivariable logistic regression analysis showed that venerable age was an independent risk factor for cognitive impairment (OR = 1.089, *P < 0.001*), in accordance with those reported in the previous papers [31, 32]. In a prospective study of 215 patients undergoing elective surgery of all types, Smith et al. found that the effect of aging on cognitive impairment was apparent. The prevalence of MCI increased with aging, with 42% of patients in the 65–69 years age group increasing to 80% of patients aged 80 years and above [32]. Currently, increasing numbers of elderly patients choose surgery to treat surgical disease [1, 33]. One or more cardiovascular and cerebrovascular diseases as well as other systemic diseases are often combined in the elderly [34]. Moreover, the coexistences of multiple preoperative medications, frailty, anxiety and depression further increase the prevalence rates of cognitive impairment and perioperative complications [35]. Univariate analysis from our data also showed higher ASA grade (*P = 0.004*) and CCI score (*P = 0.048*) in the cognitive impairment group when compared with the normal group.

There are studies which support mild cognitive impairment is related to the genetics [36]. Those who have a parent, brother or sister with Alzheimer’s are more likely to develop the disease. The risk increases if more than one family member has the illness. Therefore, it is important to investigate the family history of patients at high risk for cognitive impairment. The way of oral inquiry was used to obtain a family history of the nervous system, nevertheless, the results were almost negative. It is not possible according to its epidemiological investigation. The reason, we supposed, was a large number of patients with undiagnosed and unrecognized [8]. Therefore, we did not analyze the family history of neurological disease in these elderly.

The impact of gender on cognitive dysfunction has been a concern, while the results have varied in different studies. Lee et al. found a gender disparity in cognitive function in India. Compared with male, Indian women have poor cognitive function in their later years [37]. In contrast, the cognitive function status of women in developed countries is not significantly different from that of men, and females often have better status [38]. Evidence-based analysis indicates that gender has an impact on cognitive impairment in elderly patients, which, might be interfered by differences in BMI, tobacco and alcohol use, social and economic activity in different regions, educational attainment, and discrimination against women [37, 39]. The role of gender in cognitive function requires a multicentered study with a larger sample to confirm because of the large clinical heterogeneity.

The degree of education has a great impact on cognitive function. Studies have shown that good education and cultural background have a positive effect on the ability of concept formation, vocabulary expression, spatial structure perception and memory, while cultural restriction may contribute to a negative effect [40]. Highly educated people often have a high reserve of neurons [41]. The more people receive education, the better subjective initiative and ability to adapt to the external environment, which may stimulate brain cells [42]. The numbers of nerve connections (neurons) and information hubs (synapses) are likely to be greater in people who are highly educated. Alternatively, even if the quantity of neurons and synapses is no different, the synapses are likely to be more efficient and/or alternative circuitry is likely to be operating in those who are highly educated. Cognitive reserve is an emerging dynamic concept and is thought to be modifiable in keeping with the concept of brain plasticity [10]. A recent clinical study demonstrated that preoperative cognitive reserve might have protective effects on long-term cognitive function after surgery [43].

Atherosclerosis was an independent risk factor for cognitive impairment in our study. Most epidemiological studies have shown that vascular risk factors such as diabetes as well as increased blood glucose level, hypertension and hyperlipidemia are closely related to cognitive impairment [44, 45]. Nevertheless, the results of the present study showed that there were no differences in diabetes, hypertension and hyperlipidemia between patients with and without cognitive impairment (*P > 0.05*). There is a possibility that disease severity and the interventions subjects received are not the same. Whether nonpharmacological treatment or pharmacological therapy, the justification for treatment and the targets of management depend upon severity of the disease and the degree of organ damage [46, 47]; while not all patients would get treatment goals. Future clinical research design should filter the enrolled subjects strictly, expand the sample size, and use subgroup analysis to explore the effects of these comorbidities and their intervention on cognitive function.

A high ASA physical status is associated with substantive functional limitations in the elderly. Our study demonstrated that higher ASA score was not independent risk factors for cognitive impairment in all participants after adjusted for cofounders such as age, diabetes, therosclerosis. The explanation could be that the higher ASA score is a result of one or more moderate to severe diseases people have suffered, such as poorly controlled diabetes or hypertension, history of transient ischemic attack or coronary artery disease /stents.

Growing preclinical and clinical studies have reported associations between elevated plasma homocysteine and brain degeneration, including subtle age-related cognitive decline, cerebrovascular disease, vascular dementia, and Alzheimer disease [48]. A review by Esther et al. revealed a positive trend between cognitive decline and increased plasma Hcy concentrations in the general population and in patients with cognitive impairment [49]. Homocysteine is produced in all cells, and mechanisms of homocysteine-induced cognitive impairment include neurotoxicity and vascular injury. Some studies have suggested that protein homocysteinylation contributes to neurotoxicity, while others have shown that homocysteine induces cellular damage via oxidative stress, as well as disrupts astrocytic end-feet [48, 50]. Animal models have shown that high plasma levels of homocysteine contribute to changes in the ultrastructure of cerebral capillaries, endothelial injury, pericyte swelling, basement membrane thickening and fibrosis [51]. In keeping with the literature, patients in the cognitive impairment group had a higher level of homocysteine, even though a multivariable regression model did not find the difference.

Sleep disorders are quite common in the elderly and are mostly associated with neurodegenerative processes [52]. Moreover, sleep disorders and cognitive impairment often coexist and interact with one another in the early stages of Alzheimer’s disease [53, 54]. Sleep disorders in patients with MCI are associated with changes in memory and execution, suggesting that sleep dysfunction may be a precursor to cognitive changes [53]. The structure of sleep and EEG findings may also be abnormal, even in the early stage of MCI [53, 54]. In our study, the elderly often complained of sleep disruption due to frequent nocturia, or easy or early awakening. Electroencephalo-graph (EEG) studies also show that such patients have reduced nighttime slow wave sleep, a weakened sleep promotion process and an enhanced wakefulness process [55]. Altered sleep seriously affects normal sleep patterns: patients frequently recounted that they were sleepy in the daytime, and several rapid-eye-movement sleep episodes were exhibited in EEG during their naps [56]. In this study, compared with the normal group, the subjective sleep quality of the impairment group was poorer.

### Limitations

This study has several important limitations. One is that MMSE, the most widely used cognitive screening test, is affected by significant ceiling effects and has insufficient sensitivity for detecting MCI and mild dementia, especially in individuals with higher education levels [20, 57]. Montreal Cognitive Assessment (MoCA) can be used instead of MMSE to improve the sensitivity, with its higher requirements for health status and longer test time [58]. Another issue is that other potential confounding biases still remained. For example, anxiety during the preoperative period is the most common problem (with a prevalence of up to 80%), with a number of perioperative complications, such as an increase in cognitive dysfunction and delayed postoperative recovery [59]. We did not quantify the effect on cognition for further analysis. As risk factors for cognitive impairment, impairments in hearing and vision have an impact on perioperative complications in the elderly [60, 61]. We excluded these patients for the feasibility of assessment, which may underestimate the prevalence of preoperative cognitive impairment.

## Conclusion

Overall, our findings show that quite a few (28.61%) geriatric patients undergoing elective surgery do poorly on cognitive screening tests preoperatively, suggesting probable cognitive impairment. Patients at high risk in this population include those who are of venerable age, low education level, and have subjective sleep disorders, atherosclerosis and high cholesterol levels. Further research is necessary to consider preventive and targeted interventions in these patients.

## Data Availability

The datasets used and/or analyzed during the current study are available from the corresponding author on reasonable request.

## Abbreviations

ALB: Albumin
ALT: Alanine aminotransferase
BMI: Body mass index
CCI: Charlson Comorbidity Index
EEG: Electroencephalo-graph
FBG: Fasting blood glucose
Hcy: Homocysteine
IQR: Interquartile range
LDL: Low density lipoprotein
MCI: Mild cognitive impairment
MMSE: Mini-mental state examination
MoCA: Montreal Cognitive Assessment
SD: Standard deviation
TC: Total cholesterol
TG: Triglyceride
PND: Perioperative neurocognitive disorders
POCD: Postoperative cognitive dysfunction
POD: Postoperative delirium

## References

[1] Chow WB, Rosenthal RA, Merkow RP, Ko CY, Esnaola NF (2012). Optimal preoperative assessment of the geriatric surgical patient: a best practices guideline from the American College of Surgeons National Surgical Quality Improvement Program and the American Geriatrics Society. J Am Coll Surg.

[2] Finlayson E, Zhao S, Boscardin WJ, Fries BE, Landefeld CS, Dudley RA (2012). Functional status after colon cancer surgery in elderly nursing home residents. J Am Geriatr Soc.

[3] Beffa LR, Petroski GF, Kruse RL, Vogel TR (2015). Functional status of nursing home residents before and after abdominal aortic aneurysm repair. J Vasc Nurs.

[4] Blitz JD, Kendale SM, Jain SK, Cuff GE, Kim JT, Rosenberg AD (2016). Preoperative evaluation clinic visit is associated with decreased risk of in-hospital postoperative mortality. Anesthesiology.

[5] Amini S, Crowley S, Hizel L, Arias F, Libon DJ, Tighe P (2019). Feasibility and rationale for incorporating frailty and cognitive screening protocols in a preoperative anesthesia clinic. Anesth Analg.

[6] Plassman BL, Langa KM, Fisher GG, Heeringa SG, Weir DR, Mary Beth O (2008). Prevalence of cognitive impairment without dementia in the United States. Ann Intern Med.

[7] Hugo J, Ganguli M (2014). Dementia and cognitive impairment: epidemiology, diagnosis, and treatment. Clin Geriatr Med.

[8] Partridge JS, Dhesi JK, Cross JD, Lo JW, Taylor PR, Bell R (2014). The prevalence and impact of undiagnosed cognitive impairment in older vascular surgical patients. J Vasc Surg.

[9] Tully PJ, Baune BT, Baker RA (2013). Cognitive impairment before and six months after cardiac surgery increase mortality risk at median 11 year follow-up: a cohort study. Int J Cardiol.

[10] Kassie GM, Nguyen TA, Kalisch Ellett LM, Pratt NL, Roughead EE (2017). Preoperative medication use and postoperative delirium: a systematic review. BMC Geriatr.

[11] Ravi B, Pincus D, Choi S, Jenkinson R, Wasserstein DN, Redelmeier DA (2019). Association of duration of surgery with postoperative delirium among patients receiving hip fracture repair. JAMA Netw Open.

[12] Su X, Meng ZT, Wu XH, Cui F, Li HL, Wang DX (2016). Dexmedetomidine for prevention of delirium in elderly patients after non-cardiac surgery: a randomised, double-blind, placebo-controlled trial. Lancet.

[13] Deiner S, Luo X, Lin HM, Sessler DI, Saager L, Sieber FE (2017). Intraoperative infusion of dexmedetomidine for prevention of postoperative delirium and cognitive dysfunction in elderly patients undergoing major elective noncardiac surgery: a randomized clinical trial. JAMA Surg.

[14] Li D, Liu H (2017). Cognitive function assessment should be included in preoperative evaluation. J Biomed Res.

[15] Culley DJ, Flaherty D, Reddy S, Fahey MC, Rudolph J, Huang CC (2016). Preoperative cognitive stratification of older elective surgical patients: a cross-sectional study. Anesth Analg.

[16] Mahanna-Gabrielli E, Schenning KJ, Eriksson LI, Browndyke JN, Wright CB, Evered L (2019). State of the clinical science of perioperative brain health: report from the American Society of Anesthesiologists Brain Health Initiative Summit 2018. Br J Anaesth.

[17] Association GBoCM (2015). Suggestions from Chinese experts on preoperative evaluation of elderly patients (in Chinese). Chin J Geriatr.

[18] Xue J, Li J, Liang J, Chen S (2018). The prevalence of mild cognitive impairment in China: a systematic review. Aging Dis.

[19] Luo G, Han J, Ju Q, Qiao J, Yang J, Wu C (2002). Applicability of MMSE in West China: who is more suitable (in Chinese). Chin Ment Health J.

[20] Creavin ST, Wisniewski S, Noel-Storr AH, Trevelyan CM, Hampton T, Rayment D, et al. Mini-Mental State Examination (MMSE) for the detection of dementia in clinically unevaluated people aged 65 and over in community and primary care populations. Cochrane Database Syst Rev. 2016;(1):Cd011145. 10.1002/14651858.CD011145.pub2. 10.1002/14651858.CD011145.pub2 PMC8812342 26760674

[21] Evered LA, Silbert BS, Scott DA, Maruff P, Ames D, Choong PF (2011). Preexisting cognitive impairment and mild cognitive impairment in subjects presenting for total hip joint replacement. Anesthesiology.

[22] Lee YS, Kim YB, Lee SH, Park YS, Park SW (2016). The prevalence of undiagnosed presurgical cognitive impairment and its postsurgical clinical impact in older patients undergoing lumbar spine surgery. J Korean Neurosurg Soc.

[23] Adogwa O, Elsamadicy AA, Sergesketter A, Vuong VD, Moreno J, Cheng J (2018). Independent association between preoperative cognitive status and discharge location after surgery: a strategy to reduce resource use after surgery for deformity. World Neurosurg.

[24] Francisco José TS, Ángel B-V, Eduardo RD, Enmanuel SM, David CP, Juan Ramón DP (2014). Severity of cognitive impairment as a prognostic factor for mortality and functional recovery of geriatric patients with hip fracture. Geriatr Gerontol Int.

[25] Evered L, Silbert B, Knopman DS, Scott DA, DeKosky ST, Rasmussen LS (2018). Recommendations for the nomenclature of cognitive change associated with anaesthesia and surgery-2018. Br J Anaesth.

[26] Evered L, Silbert B, Knopman DS, Scott DA, DeKosky ST, Rasmussen LS (2018). Recommendations for the nomenclature of cognitive change associated with anaesthesia and surgery-20181. J Alzheimers Dis.

[27] Evered L, Silbert B, Knopman DS, Scott DA, DeKosky ST, Rasmussen LS (2018). Recommendations for the nomenclature of cognitive change associated with anaesthesia and surgery-2018. Anesthesiology.

[28] Evered L, Silbert B, Knopman DS, Scott DA, DeKosky ST, Rasmussen LS (2018). Recommendations for the nomenclature of cognitive change associated with anaesthesia and surgery-2018. Anesth Analg.

[29] Evered L, Silbert B, Knopman DS, Scott DA, DeKosky ST, Rasmussen LS (2018). Recommendations for the nomenclature of cognitive change associated with anaesthesia and surgery-2018. Acta Anaesthesiol Scand.

[30] Evered L, Silbert B, Knopman DS, Scott DA, DeKosky ST, Rasmussen LS (2018). Recommendations for the nomenclature of cognitive change associated with anaesthesia and surgery-2018. Can J Anaesth.

[31] Murman DL (2015). The impact of age on cognition. Semin Hear.

[32] Smith NA, Yeow YY (2016). Use of the Montreal Cognitive Assessment test to investigate the prevalence of mild cognitive impairment in the elderly elective surgical population. Anaesth Intensive Care.

[33] Subramaniyan S, Terrando N (2019). Neuroinflammation and perioperative neurocognitive disorders. Anesth Analg.

[34] Izzo C, Carrizzo A, Alfano A, Virtuoso N, Capunzo M, Calabrese M, et al. The impact of aging on cardio and cerebrovascular diseases. Int J Mol Sci. 2018;19(2). 10.3390/ijms19020481. 10.3390/ijms19020481 PMC5855703 29415476

[35] Badgwell B, Stanley J, Chang GJ, Katz MH, Lin HY, Ning J (2013). Comprehensive geriatric assessment of risk factors associated with adverse outcomes and resource utilization in cancer patients undergoing abdominal surgery. J Surg Oncol.

[36] Armstrong RA (2019). Risk factors for Alzheimer’s disease. Folia Neuropathol.

[37] Lee J, Shih R, Feeney K, Langa KM (2014). Gender disparity in late-life cognitive functioning in India: findings from the longitudinal aging study in India. J Gerontol B Psychol Sci Soc Sci.

[38] Langa KM, Larson EB, Karlawish JH, Cutler DM, Kabeto MU, Kim SY (2008). Trends in the prevalence and mortality of cognitive impairment in the United States: is there evidence of a compression of cognitive morbidity?. Alzheimers Dement.

[39] Carmel S (2019). Health and well-being in late life: gender differences worldwide. Front Med (Lausanne).

[40] Ardila A, Moreno S (2001). Neuropsychological test performance in Aruaco Indians: an exploratory study. J Int Neuropsychol Soc.

[41] Wada M, Noda Y, Shinagawa S, Chung JK, Sawada K, Ogyu K (2018). Effect of education on Alzheimer’s disease-related neuroimaging biomarkers in healthy controls, and participants with mild cognitive impairment and Alzheimer’s disease: a cross-sectional study. J Alzheimers Dis.

[42] Stern Y (2012). Cognitive reserve in ageing and Alzheimer’s disease. Lancet Neurol.

[43] Saleh AJ, Tang GX, Hadi SM, Yan L, Chen MH, Duan KM (2015). Preoperative cognitive intervention reduces cognitive dysfunction in elderly patients after gastrointestinal surgery: a randomized controlled trial. Med Sci Monit.

[44] Yaffe K, Blackwell T, Kanaya AM, Davidowitz N, Barrett-Connor E, Krueger K (2004). Diabetes, impaired fasting glucose, and development of cognitive impairment in older women. Neurology.

[45] Stough C, Pipingas A, Camfield D, Nolidin K, Savage K, Deleuil S, et al. Increases in total cholesterol and low density lipoprotein associated with decreased cognitive performance in healthy elderly adults. Metab Brain Dis. 2019. 10.1007/s11011-018-0373-5. 10.1007/s11011-018-0373-5 30649667

[46] Okocha O, Gerlach RM, Sweitzer B (2019). Preoperative evaluation for ambulatory anesthesia: what, when, and how?. Anesthesiol Clin.

[47] Vázquez-Narváez KG, Ulibarri-Vidales M (2019). The patient with hypertension and new guidelines for therapy. Curr Opin Anaesthesiol.

[48] Price BR, Wilcock DM, Weekman EM (2018). Hyperhomocysteinemia as a risk factor for vascular contributions to cognitive impairment and dementia. Front Aging Neurosci.

[49] Setien-Suero E, Suarez-Pinilla M, Suarez-Pinilla P, Crespo-Facorro B, Ayesa-Arriola R (2016). Homocysteine and cognition: a systematic review of 111 studies. Neurosci Biobehav Rev.

[50] Sudduth TL, Powell DK, Smith CD, Greenstein A, Wilcock DM (2013). Induction of hyperhomocysteinemia models vascular dementia by induction of cerebral microhemorrhages and neuroinflammation. J Cereb Blood Flow Metab.

[51] Shastry S, Tyagi SC (2004). Homocysteine induces metalloproteinase and shedding of beta-1 integrin in microvessel endothelial cells. J Cell Biochem.

[52] Pace-Schott EF, Spencer RM (2011). Age-related changes in the cognitive function of sleep. Prog Brain Res.

[53] Naismith SL, Rogers NL, Hickie IB, Mackenzie J, Norrie LM, Lewis SJ (2010). Sleep well, think well: sleep-wake disturbance in mild cognitive impairment. J Geriatr Psychiatry Neurol.

[54] Zhao C, Noble JM, Marder K, Hartman JS, Gu Y, Scarmeas N (2018). Dietary patterns, physical activity, sleep, and risk for dementia and cognitive decline. Curr Nutr Rep.

[55] Putilov AA, Munch MY, Cajochen C (2013). Principal component structuring of the non-REM sleep EEG spectrum in older adults yields age-related changes in the sleep and wake drives. Curr Aging Sci.

[56] Cagnin A, Fragiacomo F, Camporese G, Turco M, Busse C, Ermani M (2017). Sleep-wake profile in dementia with lewy bodies, Alzheimer’s disease, and normal aging. J Alzheimers Dis.

[57] O'Bryant SE, Humphreys JD, Smith GE, Ivnik RJ, Graff-Radford NR, Petersen RC (2008). Detecting dementia with the mini-mental state examination in highly educated individuals. Arch Neurol.

[58] Trzepacz PT, Hochstetler H, Wang S, Walker B, Saykin AJ, Alzheimer’s Disease Neuroimaging I (2015). Relationship between the Montreal Cognitive Assessment and Mini-mental State Examination for assessment of mild cognitive impairment in older adults. BMC Geriatr.

[59] McCleane GJ, Cooper R (1990). The nature of pre-operative anxiety. Anaesthesia.

[60] Gajdos C, Kile D, Hawn MT, Finlayson E, Henderson WG, Robinson TN (2015). The significance of preoperative impaired sensorium on surgical outcomes in nonemergent general surgical operations. JAMA Surg.

[61] Chen SP, Bhattacharya J, Pershing S (2017). Association of vision loss with cognition in older adults. JAMA Ophthalmol.

